# Investigating Developmental Status of Children Aged 0–5 Years and Its Association With Child Gender, Family Background and Geographic Locations in Australian Community‐Based Early Learning Centres

**DOI:** 10.1111/cch.70097

**Published:** 2025-05-28

**Authors:** Huahua Yin, Matthew Ankers, Alicia Bell, Yvonne Karen Parry, Eileen Willis

**Affiliations:** ^1^ College of Nursing and Health Sciences Flinders University Adelaide South Australia Australia; ^2^ Caring Futures Institute Flinders University Adelaide South Australia Australia; ^3^ University College London (UCL) London UK; ^4^ School of Nursing, Midwifery and Social Sciences Central Queensland University Adelaide South Australia Australia

**Keywords:** community socio‐economic status, culturally and linguistically diverse family, developmental concern, early childhood development, nurse practitioner

## Abstract

**Background:**

Early childhood plays a vital role in long‐term outcomes such as health, learning, behaviour and wellbeing. Evidence shows that developmental screening of children aged 0–5 years is currently inadequate and understanding of key factors influencing child development in the years before school remain limited. This study aimed to examine the associations between a child's age, gender, family background, remoteness of residence, community socio‐economic level and developmental status.

**Methods:**

This study analysed data from a Paediatric Nurse Practitioners and Registered Nurses‐led initiative, which offered Child Health Development Checks and referral support, for children attending Australian early learning centres from August 2022 to August 2023. The Brigance Screen III packages were used to do the child development screening, which assessed three domains for children aged 0–2 and five domains for those aged 2–5. Data from 1002 children (convenience sampling with children who attended the early learning centres) were included; univariable and multivariable logistic regression models and chi‐square tests were performed.

**Results:**

After controlling for other explanatory variables, children aged 2–3, were approximately six times more likely to have developmental concerns in language and self‐help domains (*p* < 0.05, *p* < 0.001), when compared to children aged 5. Boys were around twice as likely to have developmental concern in academic and self‐help domains (*p* < 0.01). Children from culturally and linguistically diverse backgrounds were approximately two to three times more likely to have developmental concerns in language and social–emotional domains (*p* < 0.01, *p* < 0.001). Children living in mid‐level socio‐economic communities were more than twice as likely to have developmental concerns in academic domains, when compared to children living in most advantaged areas (*p* < 0.05).

**Conclusions:**

This study suggests that male children, those from culturally and linguistically diverse backgrounds, or children from mid‐level socio‐economic communities may be at higher risk of experiencing developmental concerns.

**Summary**

•This study identifies a number of developmental concerns in children aged 0–5 years, which demonstrates the value of developmental checks, and appropriate referral, prior to school commencement.
- Boys are more likely to experience developmental concerns in academic and self‐help domains between the ages of 2–5 years.
- Children aged 2–5 years from culturally and linguistically diverse backgrounds were more likely to experience developmental concerns in language and social–emotional domains.
- Children from mid‐level socio‐economic communities were more likely to experience developmental concern in academic domains than those from the most advantaged areas.
- This study provides evidence for healthcare services and early learning centres to develop and implement tailored monitoring/support programmes and health and readiness promoting resources for children who may be more susceptible to developmental issues.

## 1 Introduction

Early childhood, commonly defined as 0–5 years of age (Kempler et al. 2022) plays a vital role in long‐term outcomes including health, learning, behaviour and wellbeing (Pearce et al. 2016; World Health Organisation [WHO] 2023). It is a foundational period for the development of language, cognitive, motor and social–emotional domains (GGI Insights 2024; Steed and Stein 2023). Worldwide, approximately one in six children experience a developmental difficulty (WHO 2020), with many children's developmental delays being diagnosed too late for optimal intervention. For example, in America, many children with developmental disabilities are not detected until they commence school (Centres for Disease Control and Prevention [CDC] 2023). Early detection of developmental issues, in the years before a child commences school, provide opportunities for timely referrals for optimal intervention (Komanchuk et al. 2023; Edwards et al. 2020). This in turn, can improve a child's developmental trajectory and even mitigate some, if not all, of the negative outcomes related to childhood developmental vulnerabilities (Parry et al. 2020; Royal Far West 2017). Several terms are used throughout academic literature to describe potential developmental issues; these include terms such as developmental difficulty/delay/issues/disability/vulnerabilities (Parry et al. 2024; Chando et al. 2020; Edwards et al. 2020; Correia et al. 2019). Although there is some overlap and difference in meaning between these terms, ‘developmental concern’ has been used in this study.

In Australia, the Australian Early Development Census (AEDC)^1^ is administered at schools, on a three‐yearly cycle. It is a generalist assessment and investigates successful transition to school to improve programming and planning for education (AEDC 2021). In 2021, the AEDC found that only 54.8% of children were on track with their development, whereas 22% of children were assessed as developmentally vulnerable in one or more domain and 11.4% were developmentally vulnerable in two or more domains (The Front Project 2022). The figures outlined by the 2021 AEDC report demonstrate that a considerable number of Australian children are starting school, with developmental vulnerabilities, and suggest the need for greater detection and interventions, to aid the reduction of these numbers, in the years before school. Various jurisdictional child and family health services across the country do provide free developmental surveillance programmes for children aged 0–5 years in Australia (Child and Family Health Service 2024). However, the uptake of services differs between jurisdictions, and services may not necessarily reach all families, despite attempts at universalism, suggesting again the need for further work in the area of detection and intervention to reach more families (McKenzie et al. 2023; Edwards et al. 2020).

In addition to the insufficient developmental screening of children aged 0–5 years, there is also a lack of evidence regarding factors that contribute to early childhood development. Previous studies have shown that various factors may affect child development, including individual characteristics such as genetics, age and gender (GGI Insights 2024; Bando et al. 2024); family factors such as parental education level, family income and household socio‐economic status, and home environment and the family's cultural background (including Indigenous and/or immigration status) (Correia et al. 2019; Edwards et al. 2020; Moriguchi and Shinohara 2019; Nampijja et al. 2018; The Front Project 2022; Wise 2013). Additionally community factors such as the surrounding socio‐economic status, remoteness level (child living in major cities, regional areas and remote areas), and community environment impact on the child's developmental status (The Front Project 2022; WHO 2020). However, despite the research, the evidence of the key factors influencing child development in the years before school remains limited and somewhat controversial (Rinaldi et al. 2023).

To help address the limitations noted above, this paper analysed data from an initiative led by paediatric nurse practitioners (NP) and registered nurses (RN), which offered families Child Health Development Checks (CHDC) and referral support for children aged 0–5 years, in a community setting in Australia, between August of 2022 and August 2023. The NP and RNs used the Brigance III diagnostic tool to capture potential developmental delays and areas of concern. This allowed for referrals to remedial services to take place well before the child entered school. Additionally, the nurses provided a comprehensive health check, plus mandatory reporting where appropriate. The CHDC initiative also collected a number of demographic details on the children being screened such as gender, postcode and culturally and linguistically diverse (CALD) status. These details, when analysed against the developmental screening results, allowed for the examination of key factors that might contribute to developmental delay. Consequently, the aim of this study was to examine potential associations between a child's age, gender, family background, remoteness of residence, community socio‐economic level and developmental status in relevant domains to better understand the factors that may influence early childhood development.

## 2 Ethics Approval

Ethics approval was obtained from Flinders University (2767) HREC. Informed consent was obtained from parents/guardians of participating children.

## 3 Methods

In outlining the methods, the setting, participants, data collection and analysis are discussed. Details are provided of the BRIGANCE III screening tool, including the rationale for collapsing some of the categories.

### 3.1 Settings and Participants

This study was part of a research project piloting the implementation and evaluation of a NP and RN‐led mobile community‐based assessment clinic, providing comprehensive health assessment and developmental screening for children attending not‐for‐profit early learning centres (Parry et al. 2024). The pilot engaged with the children's key educators in an interprofessional/interdisciplinary collaboration when screening children to help assess their developmental status. Parents were contacted directly if the children's Brigance score indicated the need for referrals or interventions. Participating early learning centres provided an appropriate location and onsite support to the nurses and the opportunity for screening activities.

### 3.2 Data Collection and Analysis

Data was collected in Australia from 20 early learning centres, across urban (17) and regional (3) centres, between August 2022 and August 2023.

#### 3.2.1 Child Developmental Screening Tool

The children were screened by a NP or RN using the age‐appropriate BRIGANCE Screen III package. This screening tool was used as it can identify speech, physical, social and emotional and cognitive delays, which may be indicative of developmental delays or disorders (Korell et al. 2024; Moodie et al. 2014; Jullien 2021; Kaiser et al. 2024). Brigance captures physical development, language development, adaptive behaviour and self‐help and social–emotional skills with assessment of academic skills/cognitive development starting at age 2 (Early Childhood Technical Assistance Centre 2020; French 2013; Moodie et al. 2014). The BRIGANCE Screen III is recognised as a valid and reliable developmental screening tool for children from infancy to school age (0 to 5 years) (French 2013; Glascoe 2002; Hansen et al. 2022; O'Donnell et al. 2023). BRIGANCE III has strong reliability, high internal consistency, high degree of test–retest reliability and high degree of interrater reliability (French 2013). The BRIGANCE III also has good content validity, highly valid internal structure and strong convergent validity (French 2013; Sheeran et al. 2021). The Brigance screening tools are used in Australian health services to assess development and the need for referral (Sheeran et al. 2021) and are easy to use within community settings (O'Donnell et al. 2023).

In this study, child developmental status was analysed and presented as two age groups due to the change in the number of domains assessed for each group and the complexity of the assessment tool. These two distinct age groups of children are based on the Brigance age‐appropriate development indices, which include:

1. The 0–2 years (up to a child's 2nd birthday) BRIGANCE III screening tools assess three domains: (1) physical development; (2) language development; (3) adaptive development including self‐help and social/emotional skills.
2. The 2–5 years (from a child's 2nd birthday) BRIGANCE III screening tools assess five domains: (1) physical development; (2) language development; (3) academic/cognitive development; (4) self‐help skills such as eating, toileting and dressing; (5) social and emotional skills such as playing and getting along with others.

- Much below average: 2nd percentile or less
- Below average: 3rd–15th percentile
- Low average: 16th–24th percentile
- Average: 25th–84th percentile
- Above average: 85th–97th percentile
- Much above average: 98th percentile or more

1. Below average (≤ 15th percentile): includes much below average, and below average, suggesting there is a developmental concern in a particular domain, the child needs further investigation/monitoring or clinical support.
2. Average (16–84 percentile): includes low average and average, suggesting the child's developmental status is on track or age appropriate.
3. Above average (≥ 85 percentile): includes above average and much above average, suggesting the child's developmental status is on track or advanced.

Child developmental status was identified by the BRIGANCE III tool using a percentile system. Original assessed scores for each domain were entered into a Brigance calculator to generate a percentile score (between 0 and 100), which was then used to identify a child's average ability level, comparable to children of the same age. The percentile scores were classified into 6 categories based on the Brigance child development screening document, as used in South Australian healthcare services (such as paediatrician and speech pathologists). These six categories included:

These categories were further collapsed into three development levels by the research team to reduce the complexity when analysing the data. The three new levels of developmental status were:

For ease of use for logistic regression analysis, the first level of developmental status ‘Below average’ was coded as 1, indicating there is a ‘developmental concern’ in a particular domain that needs further investigation/monitoring or clinical support. ‘Average’ and ‘Above average’ were coded as 0, suggesting that there are no concerns for the child's development in a particular domain and that this child was on track with their development in the corresponding domain. In addition, the total number of domains identified as a developmental concern for each child was also calculated. The Brigance screening tools has three development domains for children under 2 years, and five development domains for children aged 2–5 years old. Hence, the calculated ranges were between 0 and 3 for a child aged 0–2 and 0–5 for a child aged 2–5 years. Four cases were excluded from the calculation as these had missing percentile data in one or more domain and therefore could not be identified as having a developmental concern.

#### 3.2.2 Child Characteristics

Child demographic variables included in the study were the child's age, gender, postcode of residence, Aboriginal and Torres Strait Islander background (O'Donnell et al. 2023) and culturally and linguistically diverse family background (CALD). CALD refers to ‘Australians who are not of the mainstream English‐speaking Anglo‐Celtic group and are not Aboriginal or Torres Strait Islander’ (Abdul Rahim et al. 2024, 1). The CALD children had an on‐site educator with the same linguistic background who assisted with specific language nuances to enhance the screening process. Selection of these variables was based on previous studies (Bando et al. 2024; O'Donnell et al. 2023; Edwards et al. 2020; Pearce et al. 2016; Andriyani et al. 2023).

The child's family geographic locations were classified by postcode, to remoteness area matching, using the Australian Statistical Geography Standard (ASGC) Remoteness Areas, developed by the Australia Bureau of Statistics (2018). ASGC classifies the postcode into five categories based on the distance from urban areas and population density. These classifications comprise major cities, inner regional, outer regional, remote and very remote areas of Australia (Bryden et al. 2019; Kempler et al. 2022).

The community's socio‐economic status of residence was determined by matching postcode to Australian Bureau of Statistics (ABS) Socio‐Economic Indexes for Areas (SEIFA)‐Postal Area (POA) Index of Relative Socio‐economic Advantage and Disadvantage (IRSAD) deciles (ABS 2023a). SEIFA was considered suitable as it summarises the socio‐economic characteristics of an area by combining Census data such as income, education, employment, occupation, housing and family structure (ABS 2023a). The local areas are divided into 10 deciles based on SEIFA scores, with the lowest 10% as Decile 1 (the most disadvantaged areas) and the highest 10% as Decile 10, indicating the most advantaged areas (ABS 2023b). In this study, quintiles were used to present the child's socio‐economic level of residence, derived by adding two deciles together (1 and 2, 3 and 4, 5 and 6, 7 and 8, 9 and 10), with 1 representing the most disadvantaged areas and 5 representing the most advantaged areas (Pearce et al. 2016). The use of quintiles was employed as they are commonly used in child development studies (Pearce et al. 2016).

#### 3.2.3 Data Analysis

Overall, 1025 children participated in health and developmental screening from 22 August 2022 to 31 August 2023. Children excluded from the study included those over 6 years of age (*n* = 1), those who lacked postcode information (*n* = 4), children whose postcode could not be matched to ABS categories including remoteness and SEIFA (*n* = 2) or children who had missing data on all variables of Brigance Screen tool domains (*n* = 16). This left 1002 children for inclusion in the final data analysis (251 children in 0‐ to 2‐year group and 751 children in 2‐ to 5‐year group). The child's age was further categorised as two sub‐groups for 0‐ to 2‐year group, such as (1) < 1 year; (2) ≥ 1 year and < 2 years; and four sub‐groups for 2‐ to 5‐year group, such as (1) ≥ 2 year and < 3 year; (2) ≥ 3 years and < 4 years; (3) ≥ 4 years and < 5 years; and (4) ≥ 5 years and < 6 years.

Descriptive data analysis was conducted to analyse the children's characteristics and variables for each child developmental domain. Univariable and multivariable logistic regression models were performed to determine the crude and adjusted associations between the child's characteristics and developmental concerns in each developmental domain for both 0–2‐year and 2–5‐year groups. The Adaptive domain was not included in logistic regression analysis as only six out of 251 children under 2 years of age were identified with developmental concerns. Chi‐square tests were used to examine the relationship between the child's characteristics and the numbers of domains with developmental concerns. All analysis was conducted using SPSS Statistics Version 29.0.0.2.0 (Armonk, NY: IBM Corp). Statistical significance was set at *p* < 0.05.

## 4 Results

### 4.1 Child Characteristics by Age Groups

Of the 1002 children, 25% (*n* = 251) were under 2 years of age, and 75% (*n* = 751) were aged between 2 and 5 years. The median age was 2.8 years (IQR, 1.9, 3.8), ranging from 0.4 to 5.8 years. The median age for 0–2‐year group was 1.3 years (IQR, 1.1, 1.7) and the median age for 2–5‐year group was 3.4 years (IQR 2.6, 4.2). Over half of the children were male (56.5%, *n* = 566), 2.9% (*n* = 26) identified as Aboriginal and Torres Strait Islanders, and 16.1% (*n* = 147) were from CALD families. Most children (81.2%, *n* = 814) were from major cities, whereas 11.8% (*n* = 118) were from outer regional Australia. Importantly, for this study, 40.1% of children (*n* = 402) were from areas considered to be the most disadvantaged (Quintile 1), 20.5% of children (*n* = 205) were from mid‐level socio‐economic communities (Quintile 3), and 20.3% of children (*n* = 203) were from the most advantaged areas (Quintile 5) (see Table 1).

**TABLE 1:** Child characteristics by age groups.

Child characteristics		Full sample (*n* = 1002)		0–2‐year group (*n* = 251)		2–5‐year group (*n* = 751)	
		*N*	%	*n*	%	*n*	%
Gender	Female	436	43.5%	120	47.8%	316	42.1%
	Male	566	56.5%	131	52.2%	435	57.9%
ATSI^a^	No	859	97.1%	218	97.8%	641	96.8%
	Yes	26	2.9%	5	2.2%	21	3.2%
CALD^b^	No	768	83.9%	200	87.3%	568	82.8%
	Yes	147	16.1%	29	12.7%	118	17.2%
Remoteness	Major cities of Australia	814	81.2%	205	81.7%	609	81.1%
	Inner regional Australia	70	7.0%	21	8.4%	49	6.5%
	Outer regional Australia	118	11.8%	25	10.0%	93	12.4%
Community socio‐economic status (quintile)	1 (most disadvantaged)	402	40.1%	95	37.8%	307	40.9%
	2	94	9.4%	22	8.8%	72	9.6%
	3	205	20.5%	49	19.5%	156	20.8%
	4	98	9.8%	36	14.3%	62	8.3%
	5 (most advantaged)	203	20.3%	49	19.5%	154	20.5%

*Note:* Missing values not accounted for in the analysis.

Abbreviations: ATSI = Aboriginal and Torres Strait Islander, CALD = culturally and linguistically diverse.

^a^ 117 missing data.

^b^ 87 missing data.

### 4.2 Child Developmental Status by Age Groups

Overall, developmental levels varied between domains. In the 0–2‐year group, 37.1% (*n* = 93) of children were identified as below average in the language domains, followed by physical domain, with 16.3% (*n* = 41) of children identified as below average, whereas only 2.4% (*n* = 6) of children were identified as below average in the adaptive domain (see Table S1). In the 2–5‐year group, 33.3% (*n* = 250) of children were below average in the self‐help domain, followed by 20.4% in the academic domain (*n* = 153) and 19.9% in the physical domain (*n* = 149) (see Table S2).

Developmental levels varied between sub‐groups of ages. In the 0–2‐year group, children aged 1–2 years had a higher percentage of having below average scores in the language domain (40.3%, *n* = 81) (see Table S1). In the 2–5‐year group, children aged 2–3 showed the highest percentage of below average scores in the language domain (26.9%, *n* = 75) and self‐help domain (57.3%, *n* = 160) (see Table S2).

### 4.3 Associations Between Child Characteristics and Development Concerns in Various Domains

In the 0–2‐year group, there were no statistically significant associations between child characteristics and development concerns in both physical and language domains after adjustment (*p* > 0.05). In the univariable logistic regression model, children aged 1–2 were 2.1 times more likely to have a developmental concern in a language domain compared to a child aged 0–1 (COR = 2.14 [95% CI: 1.05, 4.34], *p* = 0.035) (see Tables 2 and 3). Hosmer and Lemeshow tests showed good fitness of the models (*p* = 0.430 for physical domain and *p* = 0.862 for language domain, respectively).

**TABLE 2:** Univariable logistic regression models for the association between child characteristics and developmental concerns in different domains of children aged 0–2 (*n* = 251).

Child characteristics	Physical developmental concerns		Language developmental concerns	
	COR (95% CI)	*p*	COR (95% CI)	*p*
Age group				
< 1 (reference)				
≥ 1 and < 2	0.86 (0.38, 1.95)	0.722	2.14 (1.05, 4.34)	0.035
Gender				
Female (reference)				
Male	0.96 (0.49, 1.87)	0.892	1.36 (0.81, 2.28)	0.244
ATSI^a^				
No (reference)				
Yes	1.45 (0.16, 13.42)	0.742	2.98 (0.49, 18.23)	0.237
CALD^b^				
No (reference)				
Yes	1.18 (0.42, 3.34)	0.754	2.18 (0.99, 4.77)	0.053
Remoteness				
Major cities of Australia (reference)				
Inner regional Australia	1.23 (0.39, 3.88)	0.728	0.98 (0.39, 2.47)	0.968
Outer regional Australia	0.99 (0.32, 3.08)	0.990	0.50 (0.19, 1.32)	0.161
Community socio‐economic status				
5 (most advantaged quintile) (reference)				
4	1.82 (0.45, 7.30)	0.402	0.72 (0.28, 1.85)	0.499
3	3.26 (0.96, 11.07)	0.058	1.00 (0.44, 2.30)	1.000
2	1.78 (0.36, 8.71)	0.479	1.08 (0.38, 3.07)	0.892
1 (most disadvantaged quintile)	2.63 (0.84, 8.26)	0.098	1.43 (0.70, 2.92)	0.327

*Note:* Missing values not accounted for in the analysis.

Abbreviations: ATSI = Aboriginal and Torres Strait Islander, CALD = culturally and linguistically diverse, COR = crude odds ratio.

^a^ 28 missing data.

^b^ 22 missing data.

**TABLE 3:** Multivariable logistic regression models for the association between child characteristics and developmental concerns in different domains of children aged 0–2 (*n* = 223).

Child characteristics	Physical developmental concerns		Language developmental concerns	
	AOR (95% CI)	*p*	AOR (95% CI)	*p*
Age group				
< 1 (reference)				
≥ 1 and < 2	0.77 (0.29, 2.07)	0.609	1.94 (0.84, 4.46)	0.120
Gender				
Female (reference)				
Male	0.94 (0.44, 2.00)	0.866	1.31 (0.73, 2.33)	0.367
ATSI^a^				
No (reference)				
Yes	1.05 (0.11, 10.38)	0.968	4.02 (0.59, 27.59)	0.157
CALD^b^				
No (reference)				
Yes	0.91 (0.27, 3.01)	0.872	1.87 (0.77, 4.55)	0.170
Remoteness				
Major cities of Australia (reference)				
Inner regional Australia	0.80 (0.15, 4.30)	0.791	0.83 (0.21, 3.22)	0.787
Outer regional Australia	0.72 (0.20, 2.69)	0.630	0.79 (0.27, 2.33)	0.662
Community socio‐economic status				
5 (most advantaged quintile) (reference)				
4	1.98 (0.48, 8.14)	0.343	0.57 (0.21, 1.51)	0.254
3	3.39 (0.91, 12.71)	0.070	0.85 (0.33, 2.19)	0.737
2	2.46 (0.45, 13.49)	0.300	1.52 (0.46, 5.08)	0.493
1 (most disadvantaged quintile)	2.11 (0.61, 7.30)	0.239	0.94 (0.42, 2.09)	0.884

*Note:* Missing values not accounted for in the analysis.

Abbreviations: AOR = adjusted odds ratios, ATSI = Aboriginal and Torres Strait Islander, CALD = culturally and linguistically diverse.

^a^ 28 missing data.

^b^ 22 missing data.

For the 2–5‐year group, age, gender, CALD background and community socio‐economic status demonstrated a statistically significant association with developmental concerns in particular domains. After controlling for other explanatory variables (such as gender, ATSI, CALD background, remoteness of residence and community socio‐economic status), children aged 2–3 years were around six times more likely to be identified as having a developmental concern in a language domain (AOR = 5.47 [95% CI: 1.24, 24.16], *p* = 0.025) and self‐help domain (AOR = 6.16 [95 CI%: 2.41, 15.76], *p* < 0.001), compared to children aged between 5 and 6 years. Boys were approximately two times more likely to be identified as having a developmental concern in an academic domain (AOR = 1.76, [95% CI: 1.16, 2.68], *p* = 0.008) and self‐help domain (AOR = 1.82, [95% CI: 1.25, 2.66], *p* = 0.002). Children from CALD families were 2.38 times more likely to be identified as having a developmental concern in a language domain (AOR = 2.38 [95% CI: 1.34, 4.23], *p* = 0.003) and more than three times likely to be identified as having a developmental concern in a social/emotional domain (AOR = 3.16, [95% CI: 1.75, 5.71], *p* < 0.001). In addition, children from the mid‐level socio‐economic quintile (Quintile 3) were more than twice as likely to be identified as having a developmental concern in an academic domain (AOR = 2.16 [95% CI: 1.14, 4.09], *p* = 0.019), compared to children from the most advantaged quintile (Quintile 5). Children from Aboriginal and Torres Strait Islander backgrounds or remoteness of residence did not have statistically significant associations with developmental concerns in any domains (see Tables 4 and 5). Hosmer and Lemeshow tests showed good fitness of the models (*p* = 0.167 for physical domain, *p* = 0.132 for language domain, *p* = 0.221 for academic domain, *p* = 0.759 for self‐help domain and *p* = 0.259 for social/emotional domain, respectively).

**TABLE 4:** Univariable logistic regression models for the association between child characteristics and developmental concerns in different domains of child aged 2–5.

Child characteristics	Physical developmental concerns (*n* = 749)		Language developmental concerns (*n* = 750)		Academic developmental concerns (*n* = 750)		Self‐help developmental concerns (*n* = 751)		Social/emotional developmental concerns (*n* = 749)	
	COR (95% CI)	*p*	COR (95% CI)	*p*	COR (95% CI)	*p*	COR (95% CI)	*p*	COR (95% CI)	*p*
Age group										
≥ 2 and < 3	0.52 (0.24, 1.16)	0.109	2.85 (0.97, 8.34)	0.056	0.60 (0.28, 1.29)	0.190	4.54 (1.99, 10.34)	< 0.001	1.48 (0.43, 5.10)	0.534
≥ 3 and < 4	0.74 (0.34, 1.64)	0.463	0.74 (0.24, 2.31)	0.608	0.59 (0.27, 1.28)	0.182	1.48 (0.64, 3.40)	0.362	1.70 (0.49, 5.87)	0.401
≥ 4 and < 5	0.56 (0.25, 1.27)	0.168	0.33 (0.09, 1.16)	0.084	0.41 (0.18, 0.92)	0.031	0.16 (0.06, 0.46)	< 0.001	0.64 (0.17, 2.41)	0.508
≥ 5 and < 6 (reference)										
Gender										
Female (reference)										
Male	1.46 (1.00, 2.12)	0.049	1.34 (0.88, 2.05)	0.172	1.66 (1.14, 2.41)	0.008	1.50 (1.10, 2.05)	0.011	1.52 (0.93, 2.47)	0.094
ATSI^a^										
No (reference)										
Yes	0.96 (0.32, 2.90)	0.939	0.31 (0.04, 2.37)	0.261	0.94 (0.31, 2.85)	0.914	0.64 (0.23, 1.76)	0.385	—	—
CALD^b^										
No (reference)										
Yes	1.34 (0.83, 2.14)	0.232	3.11 (1.92, 5.02)	< 0.001	1.83 (1.17, 2.87)	0.008	1.31 (0.87, 1.97)	0.201	2.69 (1.58, 4.60)	< 0.001
Remoteness										
Major cities of Australia (reference)										
Inner regional Australia	1.71 (0.89, 3.29)	0.106	0.99 (0.43, 2.26)	0.971	1.01 (0.49, 2.08)	0.976	1.19 (0.65, 2.17)	0.578	1.94 (0.90, 4.19)	0.090
Outer regional Australia	1.17 (0.69, 2.00)	0.560	0.96 (0.51, 1.80)	0.899	1.08 (0.63, 1.84)	0.776	1.07 (0.68, 1.70)	0.765	0.93 (0.44, 1.93)	0.836
Community socio‐economic status										
5 (most advantaged quintile) (reference)										
4	0.55 (0.25, 1.22)	0.139	0.55 (0.20, 1.54)	0.254	0.68 (0.28, 1.68)	0.408	0.78 (0.41, 1.48)	0.447	0.10 (0.01, 0.75)	0.026
3	0.77 (0.44, 1.33)	0.342	1.44 (0.78, 2.65)	0.247	1.79 (1.02, 3.16)	0.044	1.13 (0.71, 1.80)	0.612	0.73 (0.37, 1.43)	0.358
2	0.58 (0.28, 1.22)	0.152	0.37 (0.12, 1.12)	0.079	1.30 (0.63, 2.69)	0.484	0.79 (0.43, 1.44)	0.434	0.64 (0.26, 1.58)	0.334
1 (most disadvantaged quintile)	0.83 (0.52, 1.33)	0.438	1.19 (0.69, 2.07)	0.530	1.56 (0.93, 2.60)	0.089	0.92 (0.61, 1.39)	0.692	0.74 (0.42, 1.32)	0.308

*Note:* Missing values not accounted for in the analysis. Variable ATSI, there were no children who had ATSI background with developmental concern in the social/emotional domain.

Abbreviations: ATSI = Aboriginal and Torres Strait Islander background, CALD = culturally and linguistically diverse, COR = crude odds ratio.

^a^ 89 missing data.

^b^ 65 missing data.

**TABLE 5:** Multivariable logistic regression models for the association between child characteristics and developmental concerns in different domains of child aged 2–5.

Child characteristics	Physical developmental concerns (*n* = 656)		Language developmental concerns (*n* = 657)		Academic developmental concerns (*n* = 657)		Self‐help developmental concerns (*n* = 658)		Social/emotional developmental concerns (*n* = 684)	
	AOR (95% CI)	*p*	AOR (95% CI)	*P*	AOR (95% CI)	*P*	AOR (95% CI)	*P*	AOR (95% CI)	*P*
Age group										
≥ 2 and < 3	0.69 (0.29, 1.68)	0.416	5.47 (1.24, 24.16)	0.025	0.64 (0.27, 1.51)	0.312	6.16 (2.41, 15.76)	< 0.001	2.47 (0.54, 11.31)	0.244
≥ 3 and < 4	1.04 (0.43, 2.48)	0.939	1.88 (0.41, 8.66)	0.417	0.84 (0.36, 1.98)	0.696	2.48 (0.96, 6.38)	0.060	3.31 (0.72, 15.23)	0.124
≥ 4 and < 5	0.68 (0.28, 1.69)	0.409	0.62 (0.12, 3.22)	0.572	0.46 (0.19, 1.13)	0.089	0.25 (0.08, 0.77)	0.016	1.18 (0.24, 5.81)	0.841
≥ 5 and < 6 (reference)										
Gender										
Female (reference)										
Male	1.35 (0.89, 2.03)	0.156	1.63 (0.98, 2.71)	0.060	1.76 (1.16, 2.68)	0.008	1.82 (1.25, 2.66)	0.002	1.49 (0.88, 2.54)	0.140
ATSI^a^										
No (reference)										
Yes	1.06 (0.34, 3.31)	0.923	0.40 (0.05, 3.15)	0.380	0.89 (0.28, 2.81)	0.844	0.75 (0.24, 2.33)	0.615	—	—
CALD^b^										
No (reference)										
Yes	1.23 (0.72, 2.10)	0.457	2.38 (1.34, 4.23)	0.003	1.61 (0.97, 2.67)	0.067	1.18 (0.70, 1.97)	0.537	3.16 (1.75, 5.71)	< 0.001
Remoteness										
Major cities of Australia (reference)										
Inner regional Australia	1.96 (0.85, 4.50)	0.112	1.03 (0.34, 3.09)	0.958	1.18 (0.50, 2.77)	0.702	0.79 (0.36, 1.72)	0.546	2.14 (0.74, 6.18)	0.160
Outer regional Australia	1.22 (0.65, 2.30)	0.536	0.74 (0.34, 1.62)	0.455	0.85 (0.46, 1.60)	0.620	0.87 (0.48, 1.58)	0.648	1.22 (0.53, 2.83)	0.637
Community socio‐economic status										
5 (most advantaged quintile) (reference)										
4	0.50 (0.22, 1.14)	0.100	0.58 (0.20, 1.69)	0.316	0.70 (0.28, 1.75)	0.445	0.78 (0.38, 1.60)	0.494	0.08 (0.01, 0.64)	0.017
3	0.67 (0.35, 1.28)	0.226	1.54 (0.74, 3.23)	0.249	2.16 (1.14, 4.09)	0.019	1.41 (0.77, 2.56)	0.266	0.58 (0.26, 1.28)	0.176
2	0.50 (0.23, 1.12)	0.091	0.43 (0.13, 1.41)	0.163	1.39 (0.64, 3.02)	0.402	0.91 (0.45, 1.83)	0.795	0.55 (0.20, 1.53)	0.251
1 (most disadvantaged quintile)	0.81 (0.48, 1.36)	0.420	1.17 (0.62, 2.23)	0.628	1.67 (0.95, 2.91)	0.074	1.01 (0.61, 1.67)	0.960	0.63 (0.33, 1.20)	0.161

*Note:* Missing values not accounted for in the analysis. Variable ATSI was not included for multivariable regression model for social/emotional domain as no children from an ATSI background had developmental concern in social/emotional domain.

Abbreviations: AOR = adjusted odds ratios, ATSI = Aboriginal and Torres Strait Islander, CALD = culturally and linguistically diverse.

^a^ 89 missing data.

^b^ 65 missing data.

### 4.4 Relationship Between Child Characteristics and Numbers of Domains With Developmental Concerns

For the 0–2‐year group, there was no statistically significant relationship between the child's characteristics and the number of domains with developmental concerns (*p* > 0.05). Approximately half (46.3%, *n* = 44) of the children from the most disadvantaged areas (Quintile 1) were identified with at least one developmental concern, whereas children from the mid‐level socio‐economic communities (Quintile 3) showed the greatest percentage of developmental concerns in two to three domains (18.4%, *n* = 9) (see Table S3).

For the 2–5‐year group, there were statistically significant relationships between age, gender, child's CALD background and the number of domains with developmental concern (*p* < 0.001, *p* = 0.002 and *p* = 0.002, respectively). The percentage of children with at least one developmental concern in the 2–3 age group (64%, *n* = 178) were much higher than other age cohorts. The percentage of boys with one to three domains identified with a developmental concern was much higher than that of girls, 46.2% (*n* = 200) versus 36.0% (*n* = 113). The percentage of children from CALD families had developmental concerns for four to five domains much higher than other children, 14.4% (*n* = 17) versus 5.5% (*n* = 31). There were no statistically significant relationships between children from Aboriginal and Torres Strait Islander backgrounds, remoteness of residence, community socio‐economic status and the number of domains with developmental concerns (*p* = 0.428, *p* = 0.606 and *p* = 0.060, respectively). However, children living in the mid‐level socio‐economic communities (Quintile 3) demonstrated the highest percentage (11.5%, *n* = 18) of developmental concerns in four to five domains (see Table S4).

## 5 Discussion

The results of this study indicated that children aged 2–3 were most at risk of developmental concerns, in particular, in the language and self‐help domains, whereas children aged 1–2 demonstrated higher developmental concern in the language development domain. The majority of children aged 1–3 years that were screened as part of the CDHC initiative that informs this study were born during the COVID‐19 pandemic. Recent studies on early childhood development indicate that children born during the pandemic were at risk of developmental delay in communication, gross motor and personal–social domains, when compared to children born before the pandemic (Giesbrecht et al. 2023). Infants born during the pandemic have also exhibited significantly reduced verbal, non‐verbal and overall reduced cognitive performance, when compared to children born pre‐pandemic (Deoni et al. 2022). The findings from these studies (Giesbrecht et al. 2023; Deoni et al. 2022) demonstrate similar developmental vulnerabilities to the children born during a similar period, in our own study. The development of children born during this period was potentially influenced by the interruptions to services, the social isolation of the child and their parents and stresses caused by the pandemic (Giesbrecht et al. 2023; Johnson et al. 2024). However, further studies that take into consideration the developmental patterns of children both pre and post the pandemic are needed to help confirm these ideas. This would also help pave the way for specific strategies focusing on improving their development and assist future generations impacted by similar disasters.

This study found that boys between the ages of 2 and 5 years were more likely to have developmental concerns in comparison to girls, particularly in the academic and self‐help domains. Our study also found a marginally significant association between gender and language development, with boys more likely than girls to have a developmental concern in the language domain. Academic literature has noted a similar trend (Bouchard et al. 2009; Lewicki et al. 2018; Bando et al. 2024), for example, Bando et al.'s (2024) study of nine countries (Brazil, Chile, Colombia, India, Indonesia, Nicaragua, Peru, Senegal and Uruguay) regarding gender differences in early child development noted that girls, aged 7–48 months, performed better than boys on all language and socio‐emotional tests in all nine countries. The authors also noted that none of the associated factors they investigated (such as socioeconomic status, family characteristics, parenting practices or health inputs) were able to account for their observed differences (Bando et al. 2024). Bando et al. (2024) posits that the explanation might be differences in biological and/or social norms expectations. Rinaldi et al. (2023) support this notion, having found that individual variability among boys can partially be explained through gender differences, whereas social expectations rather than gender could explain previous findings. The role of reading, and expressive and receptive language acquisition, along with parental engagement with young children, also impacts on language attainment in the under 5s. However, despite these suggestions, the evidence for gender impact on early language acquisition remains inconclusive (Etchell et al. 2018; Rinaldi et al. 2023), and further research is needed to better understand the role of gender or socialisation in early childhood development.

The results of this study also indicated that children from CALD backgrounds were more likely to have developmental concerns in language and social/emotional domains, further supporting previous studies emphasising the impact of CALD background on early child development (Edwards et al. 2020). A report from the 2021 AEDC revealed that children from CALD backgrounds were more likely to be developmentally vulnerable at school entry (The Front Project 2022). It maybe that children from CALD backgrounds are not proficient in English and/or that they might be refugees or asylum seekers who were exposed to trauma (Queensland Health 2019). Alternatively, they may be learning both English and their native tongue, leading to some initial delay, but then achieve bilingual capacity once both languages are mastered (Edwards et al. 2020). However, as noted by Ortiz and Cehelyk (2023), although language acquisition is usually paired with age allowing children of the same age to be compared, this is not the case for children coming from multilingual families, and no assumptions can be made about how long they have been exposed to the language used for testing.

This study found that children living in the mid‐level socio‐economic communities (Quintile 3) were more likely to have developmental concerns, a finding that differs from other studies that demonstrated that children living in the most disadvantaged areas experienced more developmental delay than other groups (The Front Project 2022; Villanueva et al. 2023). Of note, our results also indicate that around one in ten children from mid‐level socio‐economic communities had developmental concerns in four to five domains, as similarly reported in the Child Development Council Report (2020). The results suggest that more support is needed for children living in mid‐level socio‐economic communities. It is possible that families living in these areas are not eligible for social service assistance programmes or similar supports offered to those living in more disadvantaged areas where governments tend to allocate additional funding and targeted resources (NSW Govenment 2023). That said, the results should be considered with caution as family socio‐economic status and home environment were not measured in this study. Future studies need to be conducted to further explore the impact of community socio‐economic levels on child development.

It has been suggested that children from Aboriginal and Torres Strait Islander backgrounds and those living in rural and remote areas are more likely to have development delays (Cappiello and Gahagan 2009; Chando et al. 2020; The Front Project 2022). However, this study did not find any statistically significant association between child developmental status and these backgrounds or residencies. This might be attributed to the very small number of children identified as Aboriginal and Torres Strait Islander in this study and the fact that the study did not extend to remote locations. Future studies should recruit children from remote areas to better understand the impact of remoteness on development (Australia Institute of Family Studies 2011).

The study has a number of limitations. Firstly, the findings might not be generalisable for all children in the community as those participating attended a specific not for profit early learning centre, with sites often located in low socio‐economic areas. Further, children who do not attend early learning centres at all may have different outcomes. Thirdly, the study only recruited from those centres in metropolitan areas and a low number of regional locations possibly skewing findings. The impact on child development of remote or regional living may differ.

## 6 Conclusion

This study provides a better understanding of factors influencing the developmental status of children aged 0–5 in different domains, suggesting that male children, those from CALD backgrounds or children from mid‐level socio‐economic communities may be at higher risk of experiencing developmental concern. This evidence, in turn, can be utilised by healthcare services and early learning centres to develop and implement tailored monitoring/support programmes/health and readiness promoting resources for children who may be more susceptible.

## Author Contributions

**Huahua Yin:** conceptualization, methodology, software, data curation, investigation, validation, formal analysis, visualization, writing – original draft, writing – review and editing. **Matthew Ankers:** conceptualization, data curation, formal analysis, investigation, methodology, project administration, validation, writing – review and editing. **Alicia Bell:** investigation, methodology, validation, writing – review and editing. **Yvonne Karen Parry:** conceptualization, funding acquisition, investigation, methodology, project administration, resources, supervision, validation, writing – review and editing. **Eileen Willis:** conceptualization, validation, writing – review and editing.

## Ethics Statement

Ethics approval was obtained from Flinders University (2767) HREC.

## Consent

Informed consents were obtained from the child's parent/guardian.

## Conflicts of Interest

The authors declare no conflicts of interest.

## Supporting information


**Table S1** Developmental status of children aged 0–2 years (*n* = 251).

**Table S2** Developmental status of children aged 2–5 years (*n* = 751).

**Table S3** The percentage of children with different numbers of developmental concerns by child characteristics of children aged 0–2 (*n* = 251).

**Table S4** The percentage of children with different numbers of developmental concerns by child characteristics of children aged 2–5 (*n* = 747).

## Data Availability

The data that support the findings of this study are available on request from the corresponding author. The data are not publicly available due to privacy and ethical restrictions.

## Endnotes

^1^
A nationwide developmental assessment examining children's development at school commencement (start their first year of primary school aged 5 years) (AEDC 2021).
