# Supplementary material for: Investigating Developmental Status of Children Aged 0–5 Years and Its Association With Child Gender, Family Background and Geographic Locations in Australian Community‐Based Early Learning Centres
Source: Child Care Health Dev. 2025 May 28;51(4):e70097. doi: 10.1111/cch.70097 (PMC12119035; doi:10.1111/cch.70097)
Supplement: Supplementary file 1 — Table S1 Developmental status of children aged 0–2 years (n = 251). [file CCH-51-e70097-s002.docx]

**Table S1: Developmental status of children aged 0-2 years (n=251)**

| **Developmental domains** | **Developmental levels** | **<1 year (n=50)** | | **1-2 year(n=201)** | | **In total (n=251)** | |
| --- | --- | --- | --- | --- | --- | --- | --- |
|  |  | n | % | n | % | n | % |
| Physical domain | Below Average | 9 | 18.0% | 32 | 15.9% | 41 | 16.3% |
|  | Average | 35 | 70.0% | 150 | 74.6% | 185 | 73.7% |
|  | Above Average | 6 | 12.0% | 19 | 9.5% | 25 | 10.0% |
| Language domain | Below Average | 12 | 24.0% | 81 | 40.3% | 93 | 37.1% |
|  | Average | 32 | 64.0% | 111 | 55.2% | 143 | 57.0% |
|  | Above Average | 6 | 12.0% | 9 | 4.5% | 15 | 6.0% |
| Adaptive domain | Below Average | 1 | 2.0% | 5 | 2.5% | 6 | 2.4% |
|  | Average | 28 | 56.0% | 143 | 71.1% | 171 | 68.1% |
|  | Above Average | 21 | 42.0% | 53 | 26.4% | 74 | 29.5% |
