# Supplementary material for: Investigating Developmental Status of Children Aged 0–5 Years and Its Association With Child Gender, Family Background and Geographic Locations in Australian Community‐Based Early Learning Centres
Source: Child Care Health Dev. 2025 May 28;51(4):e70097. doi: 10.1111/cch.70097 (PMC12119035; doi:10.1111/cch.70097)
Supplement: Supplementary file 2 — Table S2 Developmental status of children aged 2–5 years (n = 751). [file CCH-51-e70097-s004.docx]

**Table S2: Developmental status of children aged 2-5 years (n=751)**

| **Developmental Domains** | **Developmental levels** | **≥ 2 and <3**  **(n=279)** | | **≥ 3 and <4**  **(n=240)** | | **≥ 4 and <5**  **(n=197)** | | **≥ 5 and <6**  **(n=35)** | | **In total**  **(n=751)** | |
| --- | --- | --- | --- | --- | --- | --- | --- | --- | --- | --- | --- |
|  |  | **n** | **%** | **n** | **%** | **n** | **%** | **n** | **%** | **n** | **%** |
| Physical ^a^ | Below Average | 48 | 17.3% | 55 | 22.9% | 36 | 18.4% | 10 | 28.6% | 149 | 19.9% |
|  | Average | 209 | 75.2% | 157 | 65.4% | 130 | 66.3% | 21 | 60.0% | 517 | 69.0% |
|  | Above Average | 21 | 7.6% | 28 | 11.7% | 30 | 15.3% | 4 | 11.4% | 83 | 11.1% |
| Language ^b^ | Below Average | 75 | 26.9% | 21 | 8.8% | 8 | 4.1% | 4 | 11.4% | 108 | 14.4% |
|  | Average | 192 | 68.8% | 174 | 72.5% | 61 | 31.1% | 14 | 40.0% | 441 | 58.8% |
|  | Above Average | 12 | 4.3% | 45 | 18.8% | 127 | 64.8% | 17 | 48.6% | 201 | 26.8% |
| Academic ^b^ | Below Average | 60 | 21.5% | 51 | 21.3% | 31 | 15.8% | 11 | 31.4% | 153 | 20.4% |
|  | Average | 208 | 74.6% | 156 | 65.0% | 160 | 81.6% | 21 | 60.0% | 545 | 72.7% |
|  | Above Average | 11 | 3.9% | 33 | 13.8% | 5 | 2.6% | 3 | 8.6% | 52 | 6.9% |
| Self-help | Below Average | 160 | 57.3% | 73 | 30.4% | 9 | 4.6% | 8 | 22.9% | 250 | 33.3% |
|  | Average | 111 | 39.8% | 129 | 53.8% | 159 | 80.7% | 14 | 40.0% | 413 | 55.0% |
|  | Above Average | 8 | 2.9% | 38 | 15.8% | 29 | 14.7% | 13 | 37.1% | 88 | 11.7% |
| Social-emotional ^a^ | Below Average | 34 | 12.2% | 33 | 13.8% | 11 | 5.6% | 3 | 8.6% | 81 | 10.8% |
|  | Average | 158 | 56.6% | 150 | 62.5% | 143 | 73.3% | 22 | 62.9% | 473 | 63.2% |
|  | Above Average | 87 | 31.2% | 57 | 23.8% | 41 | 21.0% | 10 | 28.6% | 195 | 26.0% |

^a^ 2 missing data ^b^ 1 missing data
