# Supplementary material for: Investigating Developmental Status of Children Aged 0–5 Years and Its Association With Child Gender, Family Background and Geographic Locations in Australian Community‐Based Early Learning Centres
Source: Child Care Health Dev. 2025 May 28;51(4):e70097. doi: 10.1111/cch.70097 (PMC12119035; doi:10.1111/cch.70097)
Supplement: Supplementary file 3 — Table S3 The percentage of children with different numbers of developmental concerns by child characteristics of children aged 0–2 (n = 251). [file CCH-51-e70097-s001.docx]

**Table S3 The percentage of children with different numbers of developmental concerns by child characteristics of children aged 0-2 (n=251)**

| **Variables** | **Age Group** | | **Gender** | | **ATSI** ^a^ | | **CALD** ^b^ | | **Remoteness** | | | **Community Socio-economic Status** | | | | |
| --- | --- | --- | --- | --- | --- | --- | --- | --- | --- | --- | --- | --- | --- | --- | --- | --- |
| **Number of developmental concerns** | <1 | ≥ 1 and <2 | Female | Male | No | Yes | No | Yes | Major cities | Inner regional | Outer regional | 1 (most disadvantaged) | 2 | 3 | 4 | 5 (most advantaged) |
| **0 ^c^** |  |  |  |  |  |  |  |  |  |  |  |  |  |  |  |  |
| n | 35 | 112 | 74 | 73 | 134 | 2 | 123 | 14 | 117 | 12 | 18 | 51 | 13 | 30 | 23 | 30 |
| % | 70.0% | 55.7% | 61.7% | 55.7% | 61.5% | 40.0% | 61.5% | 48.3% | 57.1% | 57.1% | 72.0% | 53.7% | 59.1% | 61.2% | 63.9% | 61.2% |
| ASR | 1.8 | -1.8 | 1.0 | -1.0 | 1.0 | -1.0 | 1.4 | -1.4 | -1.0 | -0.1 | 1.4 | -1.2 | 0.1 | 0.4 | 0.7 | 0.4 |
| **1 ^d^** |  |  |  |  |  |  |  |  |  |  |  |  |  |  |  |  |
| n | 9 | 64 | 31 | 42 | 62 | 2 | 57 | 10 | 64 | 5 | 4 | 29 | 6 | 10 | 11 | 17 |
| % | 18.0% | 31.8% | 25.8% | 32.1% | 28.4% | 40.0% | 28.5% | 34.5% | 31.2% | 23.8% | 16.0% | 30.5% | 27.3% | 20.4% | 30.6% | 34.7% |
| ASR | -1.9 | 1.9 | -1.1 | 1.1 | -0.6 | 0.6 | -0.7 | 0.7 | 1.6 | -0.6 | -1.5 | 0.4 | -0.2 | -1.5 | 0.2 | 1.0 |
| **2-3 ^e^** |  |  |  |  |  |  |  |  |  |  |  |  |  |  |  |  |
| n | 6 | 25 | 15 | 16 | 22 | 1 | 20 | 5 | 24 | 4 | 3 | 15 | 3 | 9 | 2 | 2 |
| % | 12.0% | 12.4% | 12.5% | 12.2% | 10.1% | 20.0% | 10.0% | 17.2% | 11.7% | 19.0% | 12.0% | 15.8% | 13.6% | 18.4% | 5.6% | 4.1% |
| ASR | -0.1 | 0.1 | 0.1 | -0.1 | -0.7 | 0.7 | -1.2 | 1.2 | -0.7 | 1.0 | -0.1 | 1.3 | 0.2 | 1.4 | -1.3 | -2.0 |
|  |  |  |  |  |  |  |  |  |  |  |  |  |  |  |  |  |
| **Statistical tests** |  |  |  |  |  |  |  |  |  |  |  |  |  |  |  |  |
| Chi-square test ^f^ | 4.037 |  | 1.217 |  |  |  | 2.267 |  |  |  |  | 9.032 |  |  |  |  |
| Fisher Exact test ^g^ |  |  |  |  | 1.833 |  |  |  | 3.781 |  |  |  |  |  |  |  |
| *P value* | 0.133 |  | 0.544 |  | 0.476 |  | 0.322 |  | 0.427 |  |  | 0.340 |  |  |  |  |
| Effect size ^h^ | 0.127 |  | 0.070 |  | 0.069 |  | 0.099 |  | 0.086 |  |  | 0.134 |  |  |  |  |

^a^ 28 missing ^b^ 22 missing

^c^ No developmental concerns were identified ^d^ Developmental concern in 1 domain ^e^ Developmental concern in 2 to 3 domains

^f^ Value of Pearson Chi-square  ^g^ Value of Fisher Exact ^h^ Value of Cramers’ V

ASR=Adjusted Standard residuals ATSI: Aboriginal and Torres Strait Islander CALD: Culturally and Linguistically Diverse
