# Supplementary material for: Investigating Developmental Status of Children Aged 0–5 Years and Its Association With Child Gender, Family Background and Geographic Locations in Australian Community‐Based Early Learning Centres
Source: Child Care Health Dev. 2025 May 28;51(4):e70097. doi: 10.1111/cch.70097 (PMC12119035; doi:10.1111/cch.70097)
Supplement: Supplementary file 4 — Table S4 The percentage of children with different numbers of developmental concerns by child characteristics of children aged 2–5 (n = 747). [file CCH-51-e70097-s003.docx]

**Table S4 The percentage of children with different numbers of developmental concerns by child characteristics of children aged 2-5 (n=747)**

| **Variables** | **Age Group** | | | | **Gender** | | **ATSI** ^a^ | | **CALD** ^b^ | | **Remoteness** | | | **Community Socio-economic Status** | | | | |
| --- | --- | --- | --- | --- | --- | --- | --- | --- | --- | --- | --- | --- | --- | --- | --- | --- | --- | --- |
| **Number of developmental concerns** | ≥ 2 and <3 | ≥ 3 and <4 | ≥ 4 and <5 | ≥ 5 and <6 | Female | Male | No | Yes | No | Yes | Major cities | Inner regional | Outer regional | 1 (most disadvantaged) | 2 | 3 | 4 | 5 (most advantaged) |
| **0 ^c^** |  |  |  |  |  |  |  |  |  |  |  |  |  |  |  |  |  |  |
| n | 100 | 124 | 138 | 18 | 183 | 197 | 326 | 11 | 293 | 53 | 315 | 23 | 42 | 152 | 35 | 76 | 40 | 77 |
| % | 36.0% | 51.7% | 71.1% | 51.4% | 58.3% | 45.5% | 51.2% | 52.4% | 52.0% | 44.9% | 52.1% | 46.9% | 45.2% | 49.5% | 48.6% | 48.7% | 65.6% | 51.0% |
| ASR | -6.3 | 0.3 | 6.6 | 0.1 | 3.4 | -3.4 | -0.1 | 0.1 | 1.4 | -1.4 | 1.3 | -0.6 | -1.2 | -0.6 | -0.4 | -0.6 | 2.4 | 0.0 |
| **1-3 ^d^** |  |  |  |  |  |  |  |  |  |  |  |  |  |  |  |  |  |  |
| n | 145 | 101 | 53 | 14 | 113 | 200 | 265 | 10 | 240 | 48 | 247 | 21 | 45 | 135 | 36 | 62 | 18 | 62 |
| % | 52.2% | 42.1% | 27.3% | 40.0% | 36.0% | 46.2% | 41.6% | 47.6% | 42.6% | 40.7% | 40.8% | 42.9% | 48.4% | 44.0% | 50.0% | 39.7% | 29.5% | 41.1% |
| ASR | 4.4 | 0.1 | -4.8 | -0.2 | -2.8 | 2.8 | -0.6 | 0.6 | 0.4 | -0.4 | -1.2 | 0.1 | 1.4 | 1.0 | 1.5 | -0.6 | -2.0 | -0.2 |
| **4-5 ^e^** |  |  |  |  |  |  |  |  |  |  |  |  |  |  |  |  |  |  |
| n | 33 | 15 | 3 | 3 | 18 | 36 | 46 | 0 | 31 | 17 | 43 | 5 | 6 | 20 | 1 | 18 | 3 | 12 |
| % | 11.9% | 6.3% | 1.5% | 8.6% | 5.7% | 8.3% | 7.2% | 0.0% | 5.5% | 14.4% | 7.1% | 10.2% | 6.5% | 6.5% | 1.4% | 11.5% | 4.9% | 7.9% |
| ASR | 3.8 | -0.7 | -3.6 | 0.3 | -1.3 | 1.3 | 1.3 | -1.3 | -3.4 | 3.4 | -0.3 | 0.8 | -0.3 | -0.6 | -2.0 | 2.3 | -0.7 | 0.4 |
|  |  |  |  |  |  |  |  |  |  |  |  |  |  |  |  |  |  |  |
| **Statistical tests** |  |  |  |  |  |  |  |  |  |  |  |  |  |  |  |  |  |  |
| Chi-square test ^f^ | 62.04 |  |  |  | 12.05 |  | 1.70 |  | 12.04 |  | 2.72 |  |  | 14.98 |  |  |  |  |
| *P value* | <0.001 |  |  |  | 0.002 |  | 0.428 |  | 0.002 |  | 0.606 |  |  | 0.060 |  |  |  |  |
| Effect size ^g^ | 0.20 |  |  |  | 0.13 |  | 0.05 |  | 0.13 |  | 0.04 |  |  | 0.10 |  |  |  |  |

^a^ 89 missing ^b^ 65 missing

^c^ No developmental concerns were identified ^d^ Developmental concerns in 1 to 3 domains ^e^ Developmental concerns in 4 to 5 domains

^f^ Value of Pearson Chi-square  ^g^ Value of Cramers’ V

ASR=Adjusted Standard residuals ATSI: Aboriginal and Torres Strait Islander CALD: Culturally and Linguistically Diverse
